# A Case of Advanced Lung Adenocarcinoma Successfully Treated by Intratumoral Injection of Tirelizumab Under Tracheoscopy

**DOI:** 10.1002/cnr2.70279

**Published:** 2025-08-08

**Authors:** Huiying Liu, Xuemao Liu, Wen Jiang, Bin Yin, Dongmei Wang, Xiaoping Yang, Wenqing Jiang

**Affiliations:** ^1^ Department of Respiratory Diseases Qingdao Haici Hospital Affiliated to Qingdao University Qingdao China

## Abstract

**Introduction:**

Lung cancer is a leading cause of cancer‐related deaths, with an annual mortality rate of nearly 1.8 million. Among its subtypes, lung adenocarcinoma is increasingly prevalent. Due to its often asymptomatic nature, most patients are diagnosed at advanced stages, missing the optimal window for surgery and resulting in a poor 5‐year survival rate.

**Case Presentation:**

We present the case of an 83‐year‐old patient with stage IV lung adenocarcinoma treated with four cycles of intravenous Tirelizumab and two cycles of intratumoral Tirelizumab via tracheoscopy. The patient achieved partial clinical remission, with tumor lesions continuing to shrink upon follow‐up by August 24, 2024. No treatment‐related adverse reactions were observed, and the patient's immune function remained normal.

**Conclusions:**

This case suggests that systemic intravenous Tirelizumab combined with tracheoscopic intratumoral injection may offer a safe and effective treatment strategy for lung adenocarcinoma, particularly for patients unsuitable for chemotherapy or surgery. However, further prospective studies and clinical trials are needed to validate these findings.

## 1 Introduction

Adenocarcinoma is the most common pathological type of lung cancer in elderly patients [1]. Due to its often asymptomatic nature, most patients are diagnosed at middle or late stages, missing the optimal window for surgical intervention and resulting in a low 5‐year survival rate [2].

The 2023 ASCO guidelines for Stage III NSCLC recommend concurrent chemotherapy and radiotherapy as the primary treatment for inoperable patients in good condition [3]. Platinum‐based dual therapy, such as carboplatin plus pemetrexed, is a first‐line chemotherapy regimen for advanced NSCLC, including lung adenocarcinoma. However, many elderly patients struggle to tolerate this regimen due to significant side effects, which can compromise therapeutic efficacy and overall survival.

The emergence of immune checkpoint inhibitors (ICPis) has revolutionized cancer treatment, offering new options through monotherapy or combination regimens. However, systemic intravenous immunotherapy is often associated with multi‐organ adverse effects [4]. This raises the question of whether local intratumoral administration of ICPis, such as PD‐1 inhibitors via tracheoscopy, could maximize therapeutic efficacy while minimizing side effects.

We present the case of an 83‐year‐old patient with lung adenocarcinoma who achieved a partial clinical response after two cycles of intratumoral Tirelizumab injection via tracheoscopy combined with four cycles of systemic intravenous therapy. Follow‐up CT scans showed continued tumor reduction through August 2024. The study was approved by the Ethics Committee of Qingdao Hospital of TCM (Qingdao Haici Hospital) (Approval No. 2020 LS [K] 045).

## 2 Methods‐Case Presentation

### 2.1 General Conditions

**FIGURE 1:**
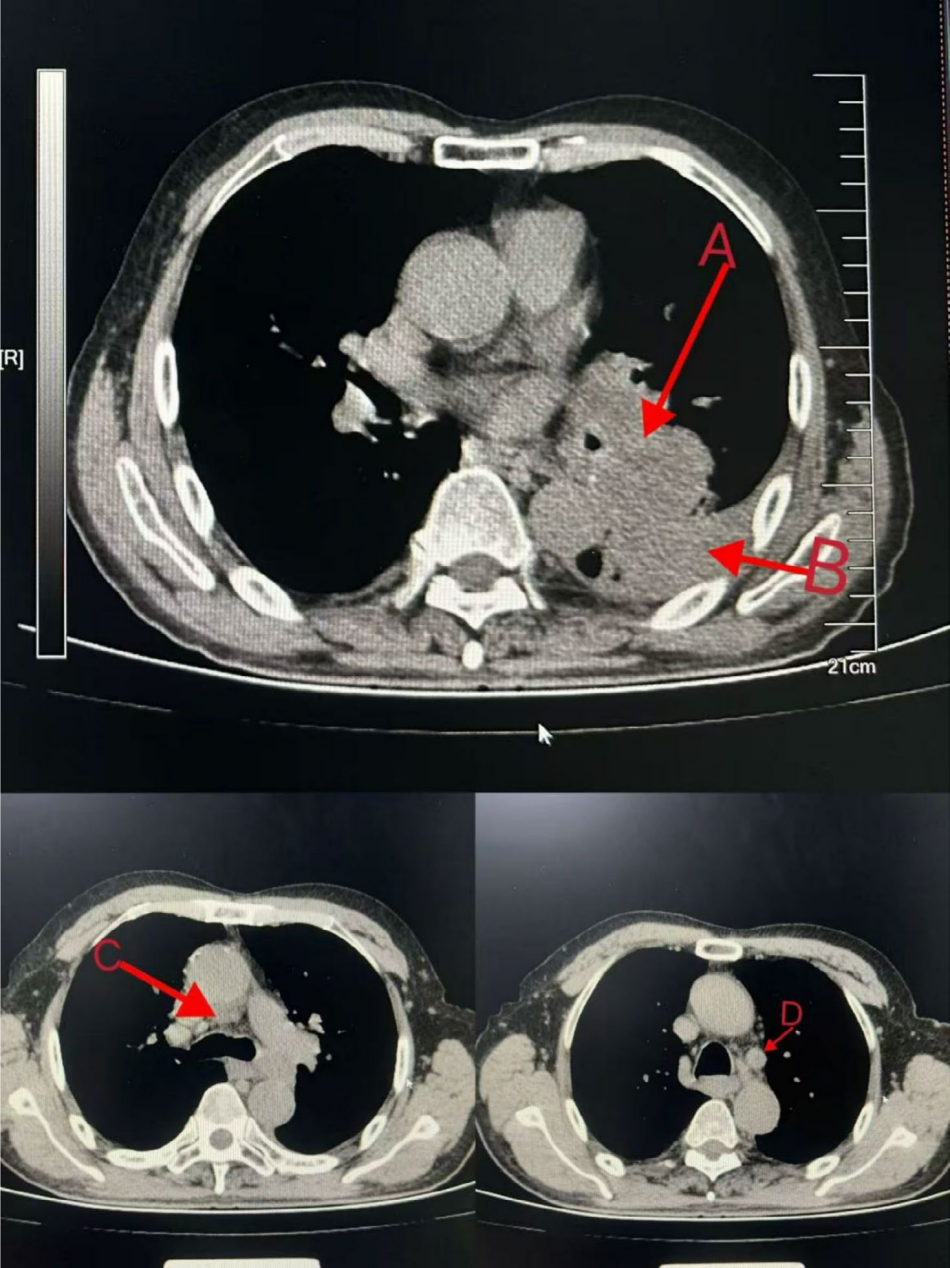
Chest CT identified a left lung mass with pleural (A) and rib invasion, multiple mediastinal (C) and hilar lymph node metastases (D), and pleural effusion (B). (2023/6/22 Chest CT).

**FIGURE 2:**
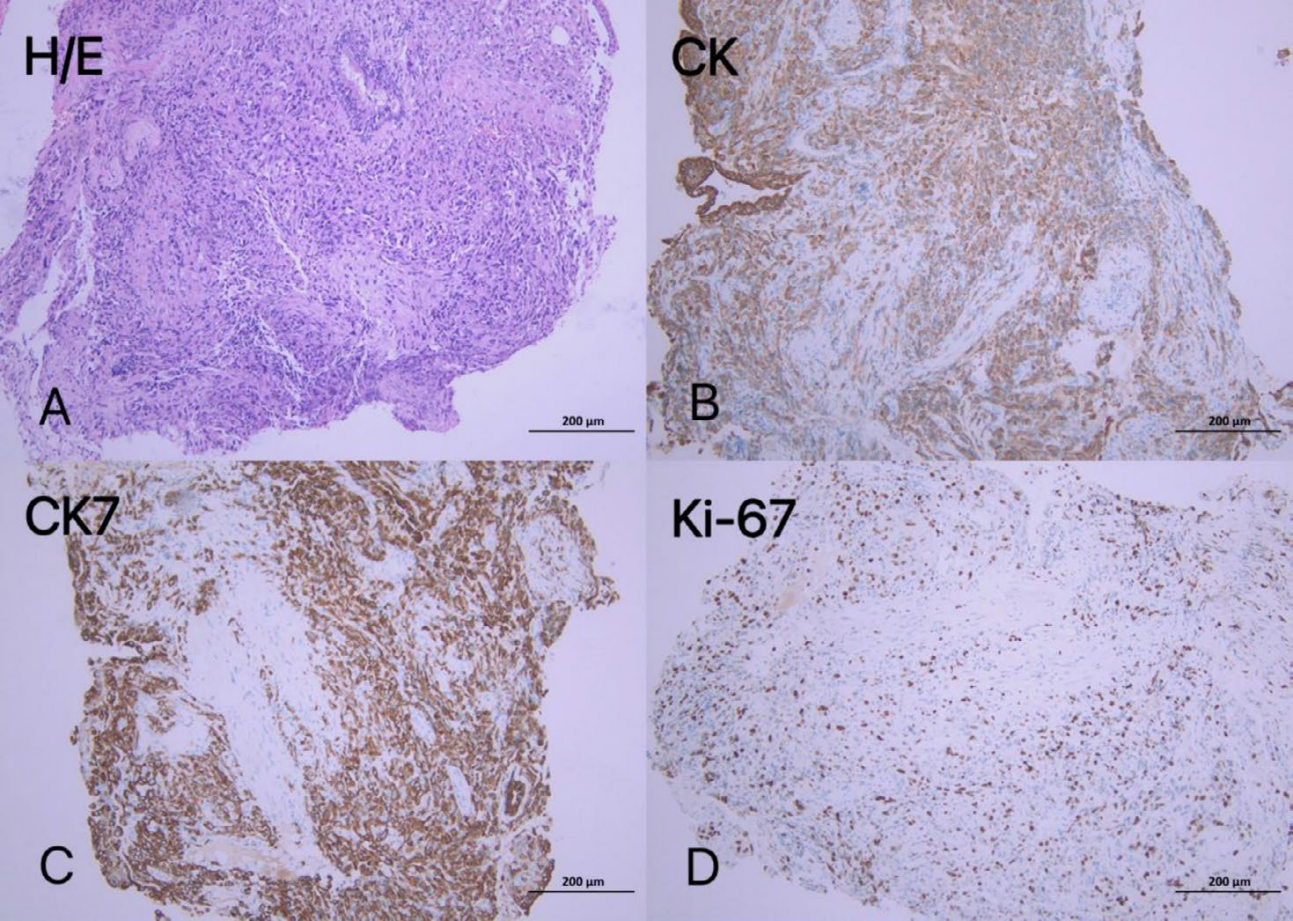
Histopathological analysis revealed the immunohistochemistry of poorly differentiated adenocarcinoma: (A) H/E; (B) CK+; (C) CK7+; (D) Ki‐67 40% + (magnification × 10). (Observation time: 2023/6).

In June 2023, an 83‐year‐old man presented to Qingdao Haici Hospital, affiliated with Qingdao University, with a 1‐week history of left flank pain. He had no significant medical history and denied smoking. Physical examination revealed diminished breath sounds in the left lung, with no superficial lymphadenopathy or cardiac abnormalities. Chest computed tomography (CT) identified a left lung mass with pleural and rib invasion, multiple mediastinal and hilar lymph node metastases, and pleural effusion (Figure 1). Abdominal enhanced CT confirmed adrenal metastases, while bone scans revealed rib metastases. Brain magnetic resonance imaging and electrocardiogram were normal. Laboratory tests showed elevated tumor markers: carbohydrate antigen CA‐125 (490 U/mL, normal 0–35 U/mL), neuron‐specific enolase (NSE) (26.1 ng/mL, normal 0–16.3 ng/mL), and cytokeratin 19 fragment assay (CA‐211) (5.20 ng/mL, normal 0–3.3 ng/mL). Routine blood tests, liver and kidney function, and cardiac indicators were within normal ranges. A bronchoscopic biopsy of the mass revealed poorly differentiated adenocarcinoma. Immunohistochemical analysis showed positivity for CK and CK7, negativity for TTF‐1 and napsin A, a Ki‐67 index of 40%, and negativity for P40 and P63 (Figure 2).

### 2.2 Diagnosis and Treatment Process

**FIGURE 3:**
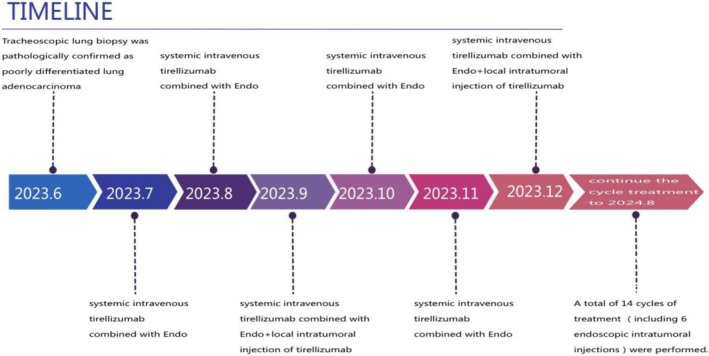
The specific treatment plan was: Tirelizumab 200 mg I.V. + Endo 30 mg I.V. once every 3 weeks. From the third treatment cycle, the patient was given local injection of Tirelizumab under bronchoscopy once every 2 treatment cycles. Follow‐up to August 24, 2014, a total of 14 cycles of treatment (including 6 endoscopic intratumoral injections) were performed. (Observation time: 2023/6–2024/8).

The patient was diagnosed with lung adenocarcinoma (T4N2aM1c, Stage IVB), with no detectable gene mutations. PD‐L1 protein expression was 100% positive in tumor cells (TC+). Given the patient's age, the family declined chemotherapy and radiotherapy. After a comprehensive evaluation of the patient's symptoms, signs, imaging results, and tumor genetic profile, and with written consent from the patient and family, a treatment plan was initiated. This included systemic intravenous Tirelizumab combined with Recombinant Human Endostatin Injection (Endo), supplemented by local intratumoral injection of Tirelizumab under bronchoscopy. The treatment regimen was as follows: Tirelizumab: 200 mg intravenously every 3 weeks (3 weeks is a treatment cycle); Endo: 30 mg intravenously on Days 1–7 of each treatment cycle; Local intratumoral injection: From the third treatment cycle, Tirelizumab (diluted to 5 mL) was administered under bronchoscopy once every two treatment cycles. The detailed treatment timeline is outlined in Figure 3.

## 3 Results

**FIGURE 4:**
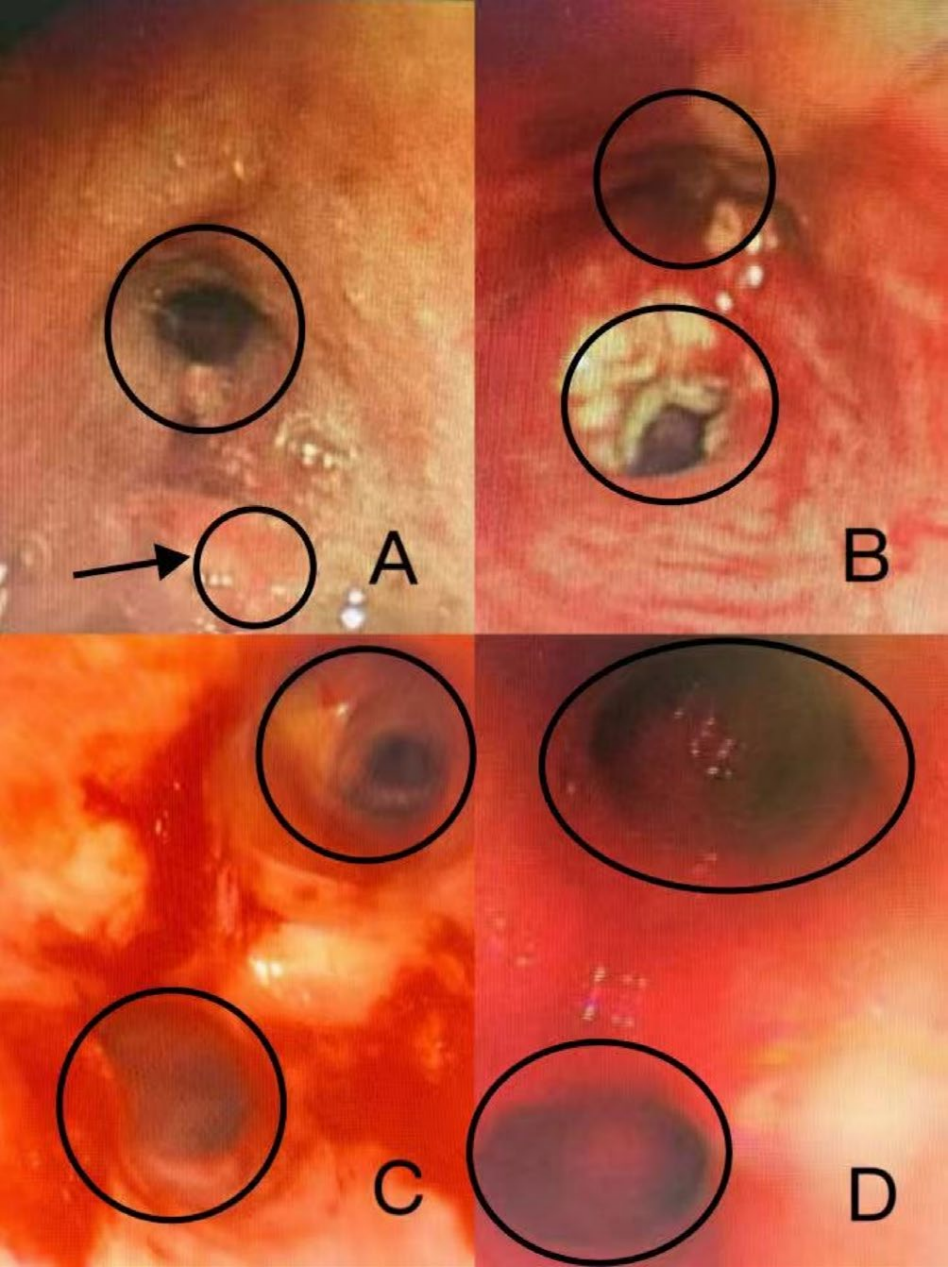
Changes under bronchoscopy. 2023/6/26 Tracheoscopy showed that the new organism completely blocked the lumen at the opening of the left lower lung lobe, and the opening of the left upper lobe of the lung was narrow (A); 2023/7/24 After freezing and thawing, the opening of the upper and lower lobes of the left lung was exposed and necrotic substances and purulent secretions were covered (B); 2023/9/22 Left upper and lower lobe opening exposed, and the lumen was narrowed (C); 2024/3/22 Full exposure of left upper and lower lobe openings (D) (Observation time: 2023/6–2024/3).

**FIGURE 5:**
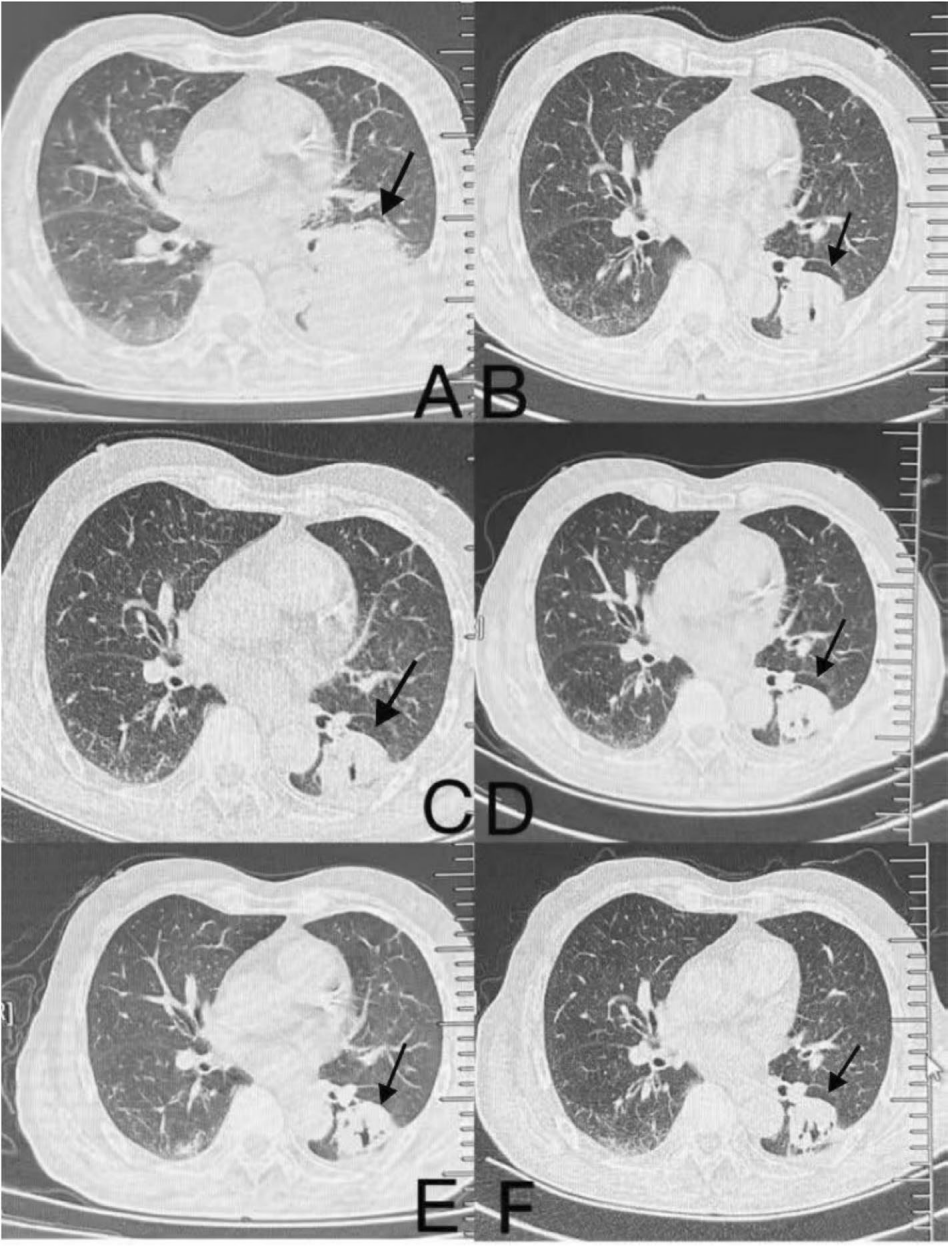
Changes under CT scan. The lung CT scan from 2023/6 to 2024/4 revealed the patient's left pulmonary mass was reduced by more than 30%, with uneven density and cavitation. 2023/6/22 Chest CT: A mass of soft tissue density shadow was observed in the lower lobe of the left lung, with uneven density and a diameter of approximately 64 mm. The adjacent bronchi were compressed and narrowed, and the pleura was invaded (A); 2023/12/22 Chest CT: A mass shadow of approximately 33 × 39 mm could be seen in the lower lobe of the left lung, with a cavity within it (B); 2024/1/22 Chest CT: A mass shadow could be seen in the lower lobe of the left lung, approximately 35 mm × 40 mm, slightly larger than before, with a cavity within it (C); 2024/2/26 Chest CT: A mass shadow of approximately 30 mm × 27 mm could be seen in the lower lobe of the left lung, with a cavity within it (D); 2024/3/21 Chest CT: A mass image of approximately 29 mm × 25 mm could be seen in the lower lobe of the left lung, with a cavity within it, and the cavity is larger than before (E); 2024/4/21 Chest CT: A mass image of approximately 26 mm × 23 mm could be seen in the lower lobe of the left lung, with a cavity within it, and the cavity is larger than before (F).

**FIGURE 6:**
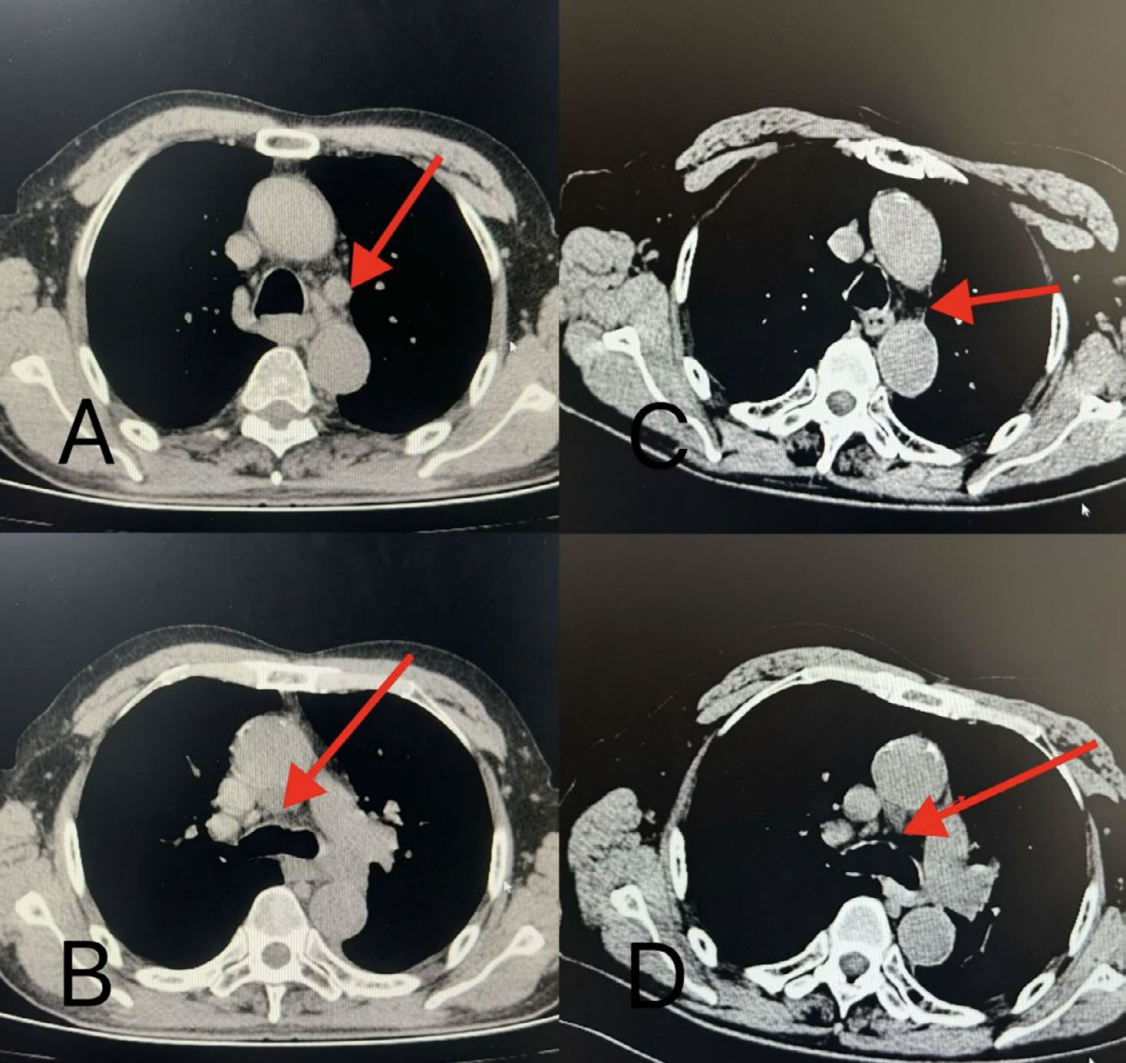
Changes of lymph nodes under CT. The lung CT scan showed that mediastinal lymph node and hilar metastasis were significantly smaller than before. 2023/6/22 Chest CT: Enlarged lymph nodes were seen beside the left hilum of the lung (A); 2023/6/22 Chest CT: Enlarged lymph nodes could be seen in the mediastinum (B); 2024/4/21 Chest CT: No significantly enlarged lymph nodes were observed beside the left hilum of the lung (C); 2024/4/21 Chest CT: No significantly enlarged lymph nodes were found in the mediastinum (D).

**FIGURE 7:**
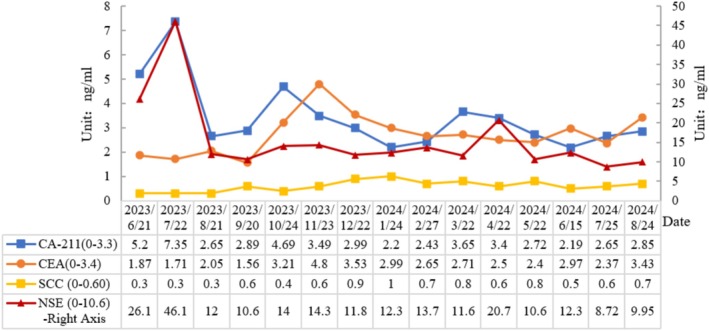
Changes in tumor indexes: CA‐211 and NSE after treatment decreased significantly compared with those before treatment, and basically stabilized at normal levels. There was no significant change in CEA and SCC before and after treatment, and the basal level was stable in the normal range. (Observation time: 2023/6–2024/8).

**FIGURE 8:**
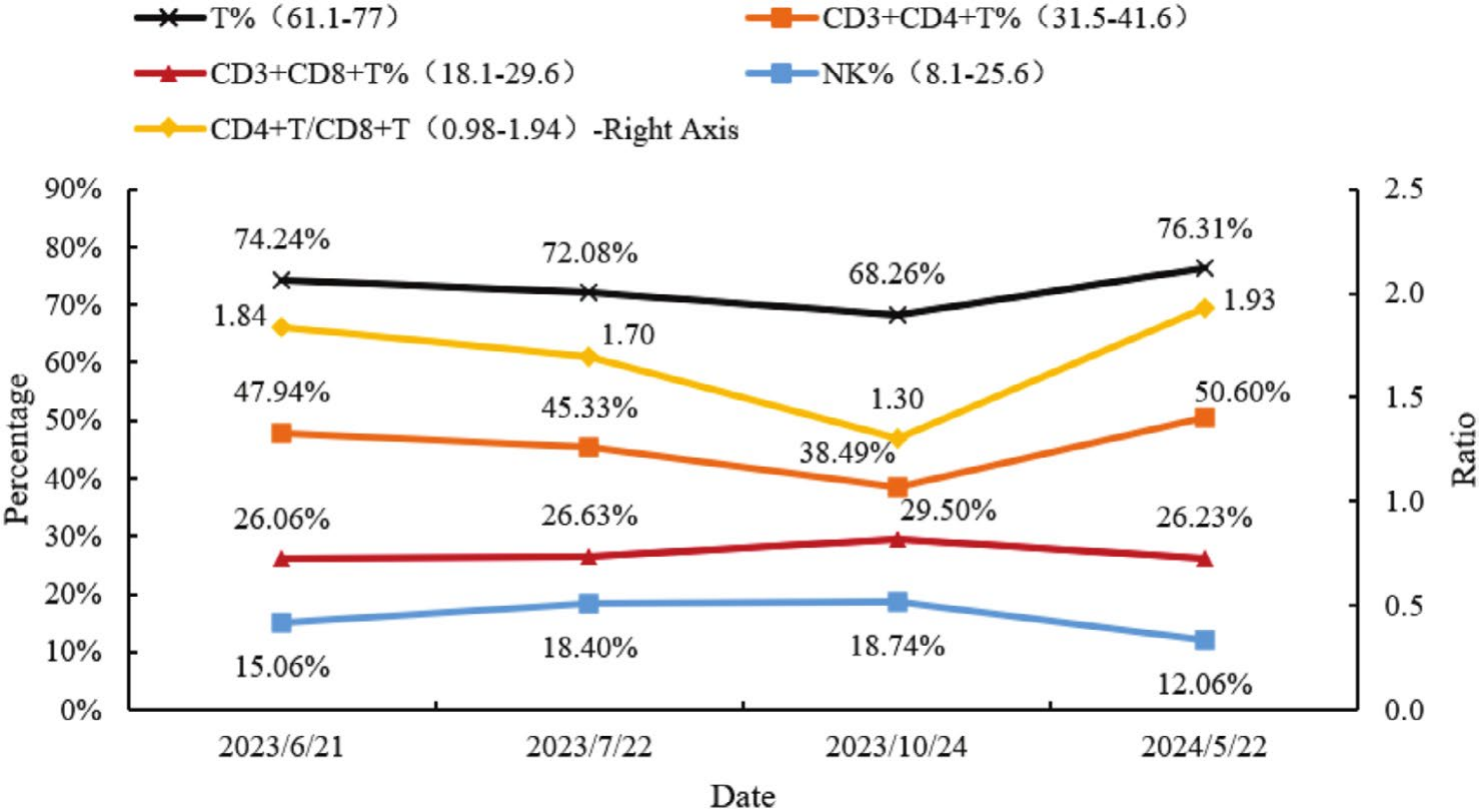
The percentage of T lymphocytes (T%), the percentage of adjuvant/induced T lymphocytes (CD3 + CD4 + T%), the percentage of inhibitory/cytotoxic T lymphocytes (CD3 + CD8 + T%), the percentage of NK cells (NK%), and the ratio of adjuvant/inhibitory T lymphocytes (CD4 + T/CD8 + T) of patients had no significant changes compared with before. (Observation time: 2023/6–2024/5).

After four cycles of treatment, including two bronchoscopic intratumoral injections, the patient's left pulmonary mass was reduced by more than 30%, and the mediastinum and hilar metastatic lymph nodes were significantly smaller than before. Bronchoscopy revealed that the left main bronchus, previously completely obstructed by the tumor, was now patent, with only mild mucosal infiltration. Follow‐up and continued treatment were conducted until August 24, completing a total of 14 cycles (including six bronchoscopic intratumoral injections). CT scan demonstrated further shrinkage of the lesions, lymph nodes in the mediastinum and hilar metastasis were significantly smaller than before, tracheoscopy showed that the left upper and lower lobe openings were completely exposed, blood tests showed that tumor markers returned to normal, and the patient's quality of life significantly improved (Figures 4, 5, 6, 7). The patient's immune function remained normal, with lymphocyte changes detailed in Figure 8. No adverse reactions or unanticipated events were observed throughout the treatment period.

## 4 Discussion

Lung cancer remains one of the leading causes of cancer‐related deaths globally, with NSCLC accounting for 85%–90% of cases [5]. For decades, platinum‐based combination chemotherapy has been the standard first‐line treatment for advanced NSCLC patients without targetable driver mutations. However, its benefits are limited, with median progression‐free survival of 4–6 months and overall survival of 10–13 months, alongside significant side effects.

Since 2009, immunotherapy has revolutionized lung cancer treatment, particularly for patients lacking oncogenic driver mutations. ICIs are humanized immunoglobulins that block molecules responsible for immune cell suppression, thereby enhancing anti‐cancer immune responses. Studies show that single‐agent immunotherapy can improve survival in over 50% of patients with PD‐L1 expression on tumor or immune cells, while offering fewer side effects compared to chemotherapy [6, 7].

However, the widespread use of ICIs has also highlighted unique immune‐related adverse events, distinct from chemotherapy toxicity [8]. These include cardiovascular, dermatological, endocrine, gastrointestinal, neurological, and pulmonary events, such as myocarditis, vitiligo, colitis, and various forms of interstitial pneumonia [9]. Balancing the efficacy of ICIs with the minimization of adverse events remains a critical clinical challenge.

Bronchoscopy, pioneered by Ikeda et al., has transformed lung cancer diagnosis and treatment [10]. Advances in equipment, navigation, and techniques have expanded its diagnostic and therapeutic potential. Over the past decade, intrabronchial tumor chemotherapy has emerged as a promising approach, delivering chemotherapy [11, 12], gene therapy [13, 14], and immune adjuvants directly to tumors via transbronchial needle injection (TBNI). These studies confirm the feasibility and safety of local injections with minimal systemic side effects.

Our department has successfully performed numerous intratumoral injection therapies, accumulating extensive experience and publishing related research. In one study, Ten NSCLC patients with central airway obstruction received intratumoral cisplatin and Endo. Post‐treatment bronchoscopy and CT scans showed significant airway widening, improved Karnofsky Performance Status (KPS), reduced shortness of breath scores, and enhanced lung function (FEV1/FVC ratio). These improvements were markedly superior to those in the control group, underscoring the potential of intratumoral injection therapy [15].

Local injection offers several theoretical advantages over systemic (intravenous) administration for cancer treatment. First, it achieves much higher local drug concentrations compared to systemic delivery. Second, it minimizes systemic side effects by reducing the drug's systemic circulation [16, 17]. Additionally, studies suggest that local intratumoral injection can target draining lymph nodes, potentially preventing metastasis [18]. However, whether local injection of immune drugs can further activate lymphocytes, enhance immune responses, or influence the tumor microenvironment and systemic immune regulatory feedback requires further investigation.

This case provides novel insights into tumor treatment, particularly for elderly patients who cannot tolerate radiotherapy, chemotherapy, or surgery. Intratumoral injection of immune drugs not only maximizes the anti‐tumor effects but also reduces the adverse reactions associated with long‐term intravenous administration, without compromising the patient's immune function.

However, this case has unique aspects. The patient's PD‐L1 expression was 100% positive in tumor cells (TC+), which may have contributed to the treatment's success. Whether this is a critical factor requires further validation. Additionally, it remains unclear whether intratumoral injection under direct tracheoscopy would benefit patients with poor immune expression. The preservation of the patient's immune function raises questions: is it due to the immune drug itself or the immune feedback triggered by intratumoral therapy?

Further clinical reports and studies are needed to address these questions, including: Which patient population is most suitable for this approach? Are there any adverse reactions associated with intratumoral immune drug injection? What is the specific mechanism of action of intratumoral immune drug injection? We look forward to more prospective studies and similar clinical cases to validate these findings.

## Author Contributions

**Huiying Liu:** data curation (lead), formal analysis (lead), investigation (lead), project administration (lead), resources lead, validation (lead), visualization (lead), writing – original draft (lead), writing – review and editing (lead). **Xuemao Liu:** data curationb (equal), formal analysis (equal), investigation (equal), methodology (equal), project administration (equal), validation (equal), visualization (equal). **Wen Jiang:** formal analysis (equal), investigation (equal), supervision (equal), validation (equal), visualization (equal). **Bin Yin:** methodology (equal), project administration (equal), resources (equal), supervision (equal), validation (equal). **Dongmei Wang:** project administration (equal), supervision (equal). **Xiaoping Yang:** conceptualization (equal), resources (equal), supervision (equal), validation (equal). **Wenqing Jiang:** conceptualization (lead), formal analysis (lead), funding acquisition (lead), investigation (equal), methodology (equal), project administration (lead), resources (lead), software (lead), supervision (lead), validation (lead), visualization (lead).

## Conflicts of Interest

The authors declare no conflicts of interest.

## Data Availability

The authors will provide relevant data upon reasonable request.
